# The ultrasound‐guided attenuation parameter is useful in quantification of hepatic steatosis in non‐alcoholic fatty liver disease

**DOI:** 10.1002/jgh3.12615

**Published:** 2021-07-16

**Authors:** Yu Ogino, Noritaka Wakui, Hidenari Nagai, Yoshinori Igarashi

**Affiliations:** ^1^ Division of Gastroenterology and Hepatology, Department of Internal Medicine (Omori) School of Medicine, Faculty of Medicine, Toho University Tokyo Japan

**Keywords:** attenuation imaging, hepatic steatosis, non‐alcoholic fatty liver disease, ultrasonography

## Abstract

**Aim:**

To determine the utility of the ultrasound‐guided attenuation parameter (UGAP) for quantifying hepatic steatosis in non‐alcoholic fatty liver disease (NAFLD).

**Methods:**

Subjects were 84 patients with NAFLD (53 men, 31 women; mean age 54 [20–81] years) who underwent liver biopsy and ultrasonography using a GE LOGIQ E9 system and C1‐6 probe at our hospital between 2017 and 2020. B‐Mode imaging of segment V in the liver was acquired and echo attenuation was assessed using UGAP. Steatosis score (S0: <5%; S1: 5%–33%; S2: 34%–66%; S3: ≥67%) from liver specimens was compared with the attenuation coefficient (AC; dB/cm/MHz) using UGAP.

**Results:**

Steatosis score was S0 for 9 patients, S1 for 40, S2 for 21, and S3 for 14. AC by steatosis score was 0.52 ± 0.07, 0.63 ± 0.07, 0.74 ± 0.06, and 0.78 ± 0.06 dB/cm/MHz for S0, S1, S2, and S3, respectively. AC by UGAP differed significantly between S0 and S1, S0 and S2, S0 and S3, S1 and S2, and S1 and S3 (all *P* < 0.01), demonstrating a significant increase with steatosis score. Receiver operating characteristic analysis showed good diagnostic performance of UGAP for patients with steatosis score ≥1, ≥2, and ≥3 (AUROC = 0.94, 0.95, and 0.88, respectively). Liver fat content (%) from liver specimens and AC (r = 0.81, *P* < 0.01) showed a significant positive correlation.

**Conclusion:**

UGAP is useful for quantifying hepatic steatosis in patients with NAFLD.

## Graphical abstract

Attenuation coefficient values obtained using ultrasound‐guided attenuation parameter could be useful for quantifying hepatic steatosis in non‐alcoholic fatty liver disease.

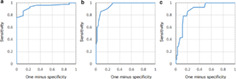

## Introduction

The number of patients with non‐alcoholic fatty liver disease (NAFLD) has been steadily growing in recent years.^1^ NAFLD affects 25.24% of the world's population and 23.71% of people in Europe, 24.13% in North America, 30.45% in South Africa, 31.79% in the Middle East, and 27.37% in Asia.^2^ It is predicted that more than 20 million people in Japan will have NAFLD by the year 2030, of which more than 1 million will have high‐risk NAFLD with stage 3 or 4 fibrosis.^3^ About 10–20% of NAFLD cases are classified as non‐alcoholic steatohepatitis (NASH), which must be diagnosed and treated proactively because it can lead to cirrhosis and liver cancer.^4^ Liver biopsy is currently the only method for definitive diagnosis of NASH,^5^, ^6^, ^7^ but it is unreasonable to routinely perform invasive liver biopsy in all patients with such a globally prevalent condition as NAFLD. The first step in identifying patients with NASH is to assess for hepatic steatosis. This is commonly done noninvasively by ultrasound examination. However, studies have shown that although B‐mode images alone are useful for assessing steatosis at levels of ≥30%,^8^ they have low sensitivity at lower levels of steatosis.^9^, ^10^ In addition, the histopathological diagnostic criteria for NASH require detection of >5% steatosis in the liver.^11^ Consequently, an accurate quantitative method for assessing hepatic steatosis is needed.

We conducted this retrospective study to determine whether a new method of attenuation imaging (ATI) using the ultrasound‐guided attenuation parameter (UGAP) would be useful for quantifying hepatic steatosis in patients scheduled to undergo liver biopsy for NAFLD.

## Methods

### Patients

Subjects were patients with NAFLD who underwent liver biopsy at Toho University Omori Medical Center between 2017 and 2020. Inclusion criteria were as follows: (i) age ≥ 16 years, and (ii) meeting the diagnostic criteria for NAFLD. NAFLD was diagnosed based on the latest guidelines established by the American Association for the Study of Liver Diseases^1^ as follows: (i) fatty change of the liver observed on imaging; (ii) no heavy alcohol consumption (ethanol intake <210 g per week for men and < 140 g per week for women); (iii) no other factors that induce fatty change of the liver; and (iv) no chronic liver disease with a clear etiology, such as viral infection (hepatitis C or hepatitis B virus), primary biliary cholangitis, or autoimmune hepatitis. Information about the study was published on the Toho University website, and patients who opted out were excluded from the study. The protocol for this retrospective study was in accordance with the Declaration of Helsinki and was approved by the ethics committee at our institution (No. M19244).

### Ultrasonography

Ultrasonography was performed from the right intercostal space using a LOGIQ E9 XDclear 2.0 ultrasound scanner (GE Healthcare) with a C1‐6‐D convex array probe. A single calibration of the ultrasound system was performed using a specific acquisition setup (fundamental B‐mode at 4.0 MHz). Images showing liver parenchyma of the right hepatic lobe (segment V) were used in the analysis. Participants were examined in the supine position with the right arm elevated above the head while breath‐holding. Patients fasted overnight before the examination. All ultrasonography examinations were performed by an independent examiner who was blinded to patient characteristics and had 25 years of experience as an ultrasonographer.

### Ultrasound‐guided attenuation parameter

We measured the attenuation coefficient (AC) of the liver using UGAP.^12^, ^13^ This method is based on comparison with a reference signal previously measured for a known attenuating material. UGAP analyses the difference between the measured liver signals and the referential signal and estimates the liver attenuation based on the difference. We set a region of interest (ROI) to avoid obvious large vessels, but the algorithm will automatically exclude small structures in the liver such as cross‐sections of the small vessels. A single operator (N.W.), who was blinded to clinical and histopathologic information, measured UGAP using a personal computer (offline) with a dedicated prototype software program in MATLAB (The Math Works, Inc., Natick, MA, USA). AC was measured twice for each patient to investigate intra‐observer variability.

### AC values and serological markers

AC values obtained for each patient by ultrasound examination were compared with the following parameters to assess correlations: serum aspartate aminotransferase (AST), alanine aminotransferase (ALT), total bilirubin, albumin, high‐density lipoprotein (HDL‐C), low‐density lipoprotein (LDL‐C), triglycerides (TG), platelet count, prothrombin time (PT%), fasting plasma glucose (FPG), and glycated hemoglobin (HbA1c). Blood samples were collected from all patients within 3 days prior to ultrasound examination.

### AC values and other parameters

Correlations of AC values with body mass index (BMI) and skin‐liver capsule distance were also assessed.

### Liver biopsy specimens

Needle biopsies were performed after sonography with a 16‐gauge liver biopsy needle (Core^II^ semiautomatic biopsy instrument; InterV Clinical Products). Specimens were obtained from the anterior segment of the right lobe (segment 5) under ultrasound guidance and fixed in 10% formalin, embedded in paraffin, sectioned, and stained with hematoxylin–eosin and Azan for histological evaluation.

Histological characteristics, NAFLD activity score, and fibrosis were evaluated using standard histological criteria by a single experienced pathologist blinded to the identity of the participants and their clinical information. The NAFLD activity score^11^ was determined based on histopathological features of steatosis (0–3), lobular inflammation (0–3), and hepatocellular ballooning (0–2). Steatosis was scored as follows: <5% = 0, 5–33% = 1, 34–66% = 2, and ≥67% = 3. Liver fat content (LFC; %) within the field of view was also determined. A single experienced pathologist examined the biopsy specimens and the ratio of fat droplet area to hepatocyte area was calculated to obtain LFC. For lobular inflammation, the scoring was as follows: no foci = 0, <2 foci = 1, 2–4 foci = 2, and > 4 foci = 3. Hepatocellular ballooning was scored as follows: none = 0, few = 1, and many = 2. Fibrosis stage was scored as follows: none = 0, mild at zone 3 = 1A, moderate at zone 3 = 1B, portal/periportal = 1C, zone 3 and periportal = 2, bridging = 3, and cirrhosis = 4.

NASH was diagnosed based on the classification described by Matteoni et al.^6^ Briefly, type 1 is defined as fatty liver alone; type 2 is defined as fat accumulation and lobular inflammation; type 3 is defined as fat accumulation and ballooning degeneration; and type 4 is defined as fat accumulation, ballooning degeneration, and either Mallory–Denk bodies or fibrosis. Type 3 or 4 is defined as NASH. Steatosis scores (S0‐3) and LFC (%) obtained by liver biopsy were compared against AC values (dB/cm/MHz) obtained by ultrasound to assess the diagnostic performance of AC values for hepatic steatosis.

### Statistical analysis

Parameter analysis according to range of AC values by steatosis score: Box plots were used to study the distribution of the range of AC values by steatosis score. Trends were evaluated using the Jonckheere–Terpstra trend test. Data were compared between the groups using the Steel–Dwass test. The diagnostic performance of AC values was assessed using receiver operating characteristic (ROC) curves. The ROC curve is a plot of the sensitivity *versus* one minus the specificity for all possible cutoff values. The most commonly used index of accuracy is the area under the receiving operating characteristic curve (AUROC), with values close to 1.0 indicating high diagnostic accuracy.

Spearman's rank correlation coefficients were used to examine the correlation of AC values with steatosis score and LFC (%) obtained by biopsy, as well as AST, ALT, total bilirubin, albumin, HDL‐C, LDL‐C, TG, platelet count, PT%, FPG, HbA1c, BMI, and skin‐liver capsule distance.

All analyses were performed using Excel Statistics 2015 software (SSRI Co., Tokyo, Japan). Differences were considered significant at *P* < 0.05.

## Results

### Patients

This study enrolled 91 patients who consented to participate. After excluding 7 patients (5 histopathologically diagnosed with a disease other than NAFLD and 2 with poor biopsy quality [shorter than 15 mm or fewer than 6 portal tracts under the microscope]), 84 patients comprising 53 men and 31 women aged 54 ± 13 (range 20–82) years were included in the analysis.

The steatosis score was S0 in 9 patients, S1 in 40 patients, S2 in 21 patients, and S3 in 14 patients. By steatosis score, AC values were 0.52 ± 0.07, 0.63 ± 0.07, 0.74 ± 0.06, and 0.78 ± 0.06 dB/cm/MHz for S0, S1, S2, and S3, respectively.

The clinical and biochemical characteristics of the 84 patients with chronic liver disease enrolled in this study are summarized in Tables 1 and 2.

**Table 1:** Clinical and biochemical characteristics

	Value^†^
Variable	NAFLD
Number	84
Sex (male/female)	53/31
Age (years)	54 ± 13
Comorbidities (HT/DM/dyslipidemia)	43/56/71
Body mass index (kg/m^2^)	29.0 ± 4.3
Skin thickness overlying the liver (mm)	21.0 ± 3.8
Aspartate aminotransferase (IU/L)	43.6 ± 22.4
Alanine aminotransferase (IU/L)	61.7 ± 41.2
Albumin (g/dL)	4.2 ± 0.4
Total bilirubin (mg/dL)	0.9 ± 0.4
Fasting plasma glucose (mg/dL)	134.0 ± 42.0
Hemoglobin A1c (%)	6.8 ± 1.3
HDL‐C (mg/dL)	50.4 ± 14.6
LDL‐C (mg/dL)	114.5 ± 33.1
Triglyceride (mg/dL)	159.5 ± 60.9
Platelet count (×10^4^/μL)	21.1 ± 6.7
Prothrombin time (% of normal)	99.5 ± 16.8

^†^ Values are expressed as the mean ± SD or numbers of patients.

DM, diabetes mellitus; HDL‐C, high‐density lipoprotein cholesterol; HT, hypertension; LDL‐C, low‐density lipoprotein cholesterol; NAFLD, non‐alcoholic fatty liver disease.

**Table 2:** Histological characteristics

	Value^†^
Variable	NAFLD
Fibrosis stage	
F0	21
F1	25
F2	7
F3	17
F4	14
Lobular inflammation (activity grade)	
A0	0
A1	67
A2	17
A3	0
Steatosis score	
S0 (<5%)	9
S1 (5–33%)	40
S2 (34–66%)	21
S3 (≥67%)	14
Hepatocellular ballooning	
B0	48
B1	32
B2	4
Liver fat content (%)	32.7 ± 24.1
NASH/not NASH	48/36

^†^ Values are expressed as the mean ± SD or numbers of patients.

NAFLD, non‐alcoholic fatty liver disease. NASH, non‐alcoholic steatohepatitis.

### Correlation between AC values and serological markers

AC values showed a significant positive correlation with ALT (*r* = 0.41, *P* < 0.01), LDL‐C (*r* = 0.33, *P* < 0.01), platelet count (*r* = 0.39, *P* < 0.01), and PT% (*r* = 0.47, *P* < 0.01), demonstrating that AC increased with increases in ALT, LDL‐C, platelet count, and PT%. There was no significant correlation of AC values with AST (*r* = 0.17, *P* = 0.12), T‐Bil (*r* = −0.16, *P* = 0.15), albumin (*r* = 0.15, *P* = 0.18), HDL‐C (*r* = −0.06, *P* = 0.65), TG (*r* = 0.20, *P* = 0.06), FPG (*r* = 0.08, *P* = 0.46), or HbA1c (*r* = 0.06, *P* = 0.61).

### Correlation between AC values and other parameters

AC values were not significantly correlated with BMI (*r* = 0.008, *P* = 0.94) or skin thickness overlying the liver (*r* = 0.06, *P* = 0.59).

### Correlation between AC values and steatosis score

**Figure 1:**
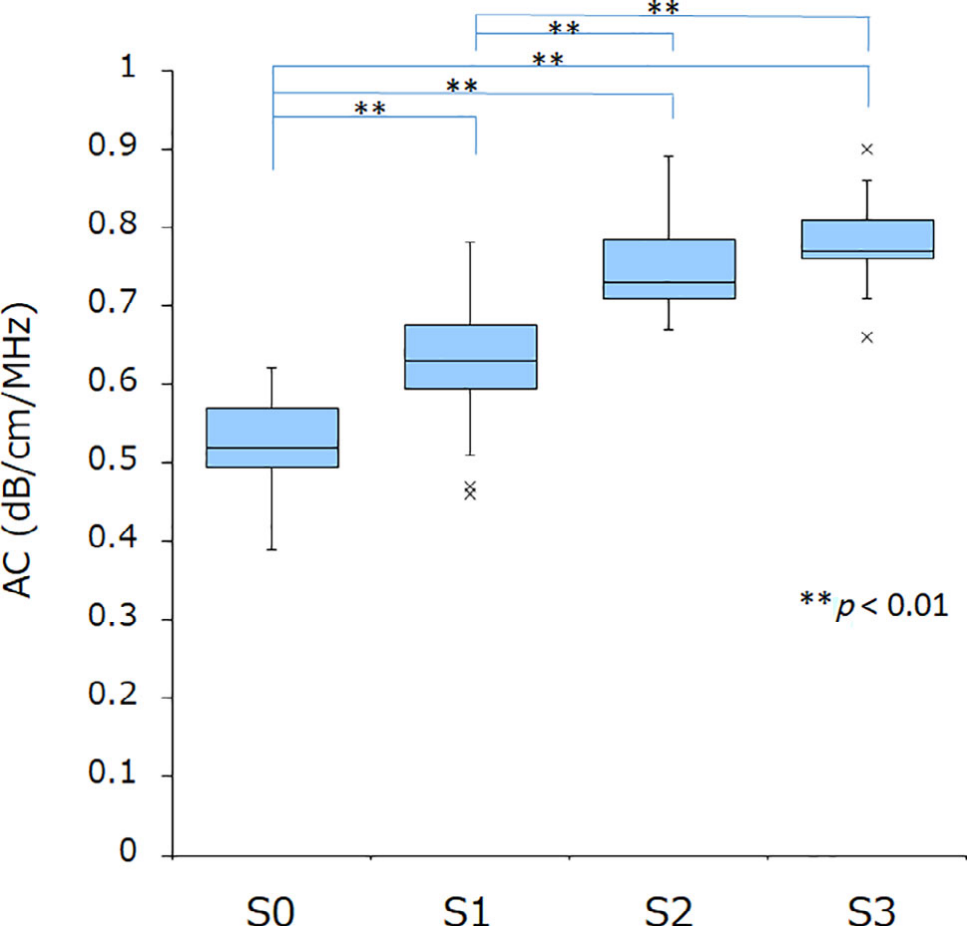
Correlation between attenuation coefficient (AC) values and steatosis score. By steatosis score, the AC value was 0.52 ± 0.07 for S0, 0.63 ± 0.0 for S1, 0.74 ± 0.06 for S2, and 0.78 ± 0.06 for S3. Multiple comparison tests showed a significant difference between S0 and S1, S0 and S2, S0 and S3, S1 and S2, and S1 and S3 (all *P* < 0.01), demonstrating that AC increased significantly with the progression of steatosis.

The AC values by steatosis score were 0.52 ± 0.07 for S0, 0.63 ± 0.0 for S1, 0.74 ± 0.06 for S2, and 0.78 ± 0.06 for S3. The Jonckheere–Terpstra trend test for strain index variation in S0–S3 patients showed a significant decreasing trend in the AC value with increasing steatosis score (*P* < 0.0001). Multiple comparisons tests showed significant differences between S0 and S1, S0 and S2, S0 and S3, S1 and S2, and S1 and S3 (all *P* < 0.01), demonstrating that AC increased significantly with the progression of steatosis (Fig. 1).

**Figure 2:**
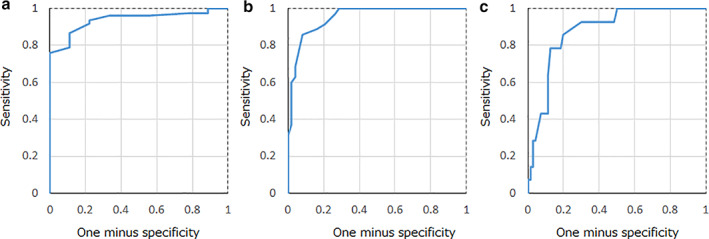
Diagnostic performance of attenuation coefficient (AC) values for liver steatosis score. By steatosis score, the cutoff value and area under receiver operating characteristic curve were respectively (a) 0.60 and 0.94 for S0 and ≥ S1, (b) 0.71 and 0.95 for S0–1 and ≥ S2, and (c) 0.72 and 0.88 for S0–S2 and ≥ S3. The sensitivity and specificity of AC values were respectively 86.7 and 88.9% for ≥S1 cases, 85.7 and 91.8% for ≥S2 cases, and 85.7 and 80.0% for ≥S3 cases.

The cutoff value and AUROC by steatosis score were respectively 0.60 and 0.94 for S0 and ≥S1, 0.71 and 0.95 for stages S0–1 and ≥S2, and 0.72 and 0.88 for S0–S2 and ≥S3. The sensitivity and specificity of AC values were respectively 86.7 and 88.9% for ≥S1 cases, 85.7 and 91.8% for ≥S2 cases, and 85.7 and 80.0% for ≥S3 cases. The positive predictive value, negative predictive value, positive likelihood ratio, and negative likelihood ratio were, respectively, 98.5, 44.4, 7.80, and 0.15 for ≥S1; 88.2, 90.0, 10.5, 0.16 for ≥S2; and 46.2, 96.6, 4.29, and 0.18 for ≥S3 (Fig. 2a–c, Table 3).

**Table 3:** Assessment of histological steatosis score based on the attenuation coefficient values in 84 patients with NAFLD

	≥S1	≥S2	≥S3
Cutoff value (mm)	0.60	0.71	0.72
Sensitivity (%)	86.7	85.7	85.7
Specificity (%)	88.9	91.8	80.0
PPV	98.5	88.2	46.2
NPV	44.4	90.0	96.6
LR+	7.80	10.5	4.29
LR−	0.15	0.16	0.18
AUROC curve	0.94	0.95	0.88

AUROC, area under receiver operating characteristic curve; LR−, negative likelihood ratio; LR+, positive likelihood ratio; NAFLD, non‐alcoholic fatty liver disease; NPV, negative predictive value; PPV, positive predictive value.

### Correlation between AC values and LFC (%)

**Figure 3:**
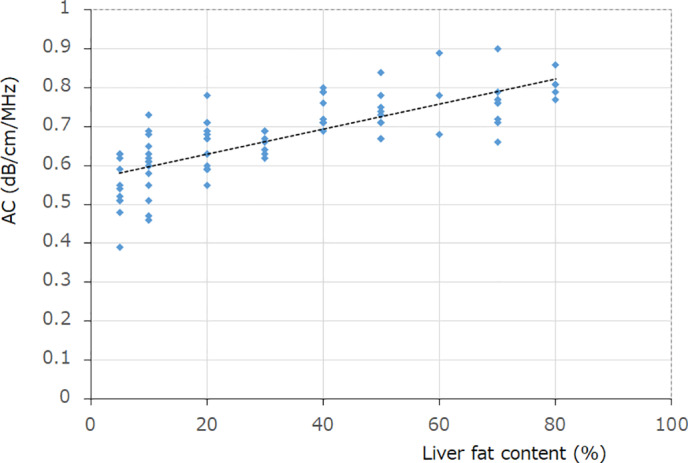
Correlation between attenuation coefficient (AC) values and liver fat content (LFC) (%). There was a significant positive correlation between LFC (%) obtained by liver biopsy and AC values (*r* = 0.81, *P* < 0.01).

There was a significant positive correlation between LFC (%) obtained by liver biopsy and AC values (*r* = 0.81, *P* < 0.01; Fig. 3).

## Discussion

The prevalence of hepatic steatosis has been increasing rapidly, and it is currently detected in over 25% of people undergoing routine health checkups in Japan.^14^ There is a long history of using ultrasound to diagnose hepatic steatosis, starting when Joseph et al. proposed the concept of a “bright liver” in 1979,^15^ and it is still often routinely used for this purpose in clinical practice. Findings such as hepatorenal echo contrast,^16^ deep attenuation, and vascular blurring were subsequently added to form a set of four characteristic features to aid in the diagnosis of this condition. However, improved beam penetration resulting from advances in ultrasound systems has altered how the characteristic features of hepatic steatosis appear on ultrasound. Specifically, deep attenuation has become difficult to capture with modern ultrasound systems because attenuation correction methods cause deep attenuation to be displayed as if there were no attenuation at all. Thus, it is actually becoming difficult to accurately assess hepatic steatosis using conventional methods alone. Also, there is now a critical need to assess whether hepatic steatosis occurs in >5% of the liver because this is the definition of steatosis used in the diagnosis of NASH, which can progress to cirrhosis.^1^ Accurate quantification of hepatic steatosis is therefore necessary, but liver biopsy has been the only quantitative method available to date. However, liver biopsy is a poorly suited diagnostic test for such a prevalent condition because of the costs, possible risks, and invasiveness of the procedure.^17^

One method currently available for quantifying steatosis is the ATI modality offered by Canon Medical Systems. Tada et al.^18^ compared ATI results against liver biopsy results in 38 patients with NAFLD using ROC curve analysis and found good diagnostic performance for steatosis scores of 1, 2, and ≥ 3 (AUROC = 0.77, 0.88, and 0.86, respectively). Bae et al.^19^ compared ATI results with liver biopsy results in 108 patients with diffuse liver disease using ROC curve analysis and also found good diagnostic performance for steatosis scores of 1, 2, and ≥3 (AUROC = 0.843, 0.886, and 0.926, respectively). Other methods have also been evaluated. Hyodo et al.^20^ evaluated the utility of computed tomography (CT) for quantifying steatosis. They compared dual‐energy CT results with liver biopsy results in 33 NAFLD patients by ROC curve analysis and found good diagnostic performance for steatosis scores of ≥1 based on AUROC. Two studies have evaluated the utility of magnetic resonance imaging (MRI). Igarashi et al.^21^ compared multi‐slice and multipoint MRI findings with liver biopsy findings in 52 patients with NAFLD by ROC curve analysis and found good diagnostic performance for steatosis scores of 1, 2, and ≥3 (AUROC = 0.975, 0.929, and 0.969, respectively). Imajo et al.^22^ compared MRI‐based proton density fat fraction results with liver biopsy results in 142 NAFLD patients by ROC curve analysis and found good diagnostic performance for steatosis scores of 1, 2, and ≥3 (AUROC = 0.98, 0.90, and 0.79, respectively).

In the present study, we tested the performance of the UGAP developed as an attenuation imaging method for quantifying relative attenuation in the liver caused by the properties of living tissues for the quantification of steatosis in patients with NAFLD. We found a significant positive correlation between LFC (%) obtained by liver biopsy and AC values (*r* = 0.81, *P* < 0.01), as well as good diagnostic performance of AC values for steatosis scores of 1, 2, and ≥3 in ROC curve analysis (AUROC = 0.94, 0.95, and 0.88, respectively). Our results indicate that ultrasound diagnosis of steatosis using UGAP has comparable performance to that previously reported for diagnosis of steatosis by ultrasound with ATI, CT, and MRI. The assessment results obtained using UGAP were favorable probably because this technology automatically detected two different positions within the ROI that were in the most suitable condition for measuring liver signals to determine attenuation.

In the 84 patients with NAFLD, AC values showed a weak positive correlation with ALT (*r* = 0.41, *P* < 0.01), platelet count (*r* = 0.39, *P* < 0.01), and PT% (*r* = 0.47, *P* < 0.01). Also, in these patients, the AC value significantly decreased with the progression of fibrosis, which is a pathological factor in the liver. When fibrosis progresses to cirrhosis, the degree of steatosis decreases (the so‐called burnout NASH), and this may be one of the possible reasons for the positive correlations of AC with PLT and PT%, and for the decreases in AC with the progression of fibrosis. The positive correlation between AC values and ALT suggests that the degree of liver inflammation influences the AC value, but this needs to be investigated further in the future. The AC value showed no significant correlation with obesity‐ and diabetes‐related factors such as BMI, FPG, and HbA1c. Measurement of UGAP may enable measurement of the liver fat amount without being affected by these three factors. This needs to be investigated in detail in the future.

Previous studies have shown that the level of hepatic steatosis correlates with cardiovascular events,^23^ and that steatosis ≥25% clearly worsens the prognosis for liver transplantation.^24^ Therefore, accurate quantification of hepatic steatosis is crucial, and noninvasive tools such as UGAP would be useful in clinical practice. Distinguishing NASH cases from NAFLD cases is extremely important, but presently liver biopsy is the only method for doing so. However, in the future, it may be possible to detect NASH in a noninvasive manner by combining a hepatic steatosis index (obtained by UGAP) with a liver fibrosis index (obtained by elastography) and certain markers in the blood. This needs to be investigated in future.

Our study has some limitations. This was a single‐center study with a small sample size, and the results will need to be validated in a multicenter study with a larger sample size. Research in different racial groups will also need to be conducted because we evaluated only Japanese patients in this study. We also did not evaluate the reproducibility (inter‐observer variability) for measuring AC values using UGAP.

Taken together, our findings indicate that AC values obtained using UGAP could be a useful new method for quantifying steatosis in NAFLD.

## Patient consent

Information about the study was published on the Toho University website, and patients who opted out were excluded from the study.

## Data availability statement

Due to the nature of this research, participants of this study did not agree for their data to be shared publicly, so supporting data are not available.
